# Magnetic resonance imaging in patients with cardiac pacemakers: era of "MR Conditional" designs

**DOI:** 10.1186/1532-429X-13-63

**Published:** 2011-10-27

**Authors:** Jerold S Shinbane, Patrick M Colletti, Frank G Shellock

**Affiliations:** 1 Division of Cardiovascular Medicine/Cardiovascular and Thoracic Institute, Keck School of Medicine, University of Southern California, Los Angeles, CA 90033, USA; 2 Department of Radiology, Keck School of Medicine, University of Southern California, Los Angeles, CA90033, USA

## Abstract

Advances in cardiac device technology have led to the first generation of magnetic resonance imaging (MRI) conditional devices, providing more diagnostic imaging options for patients with these devices, but also new controversies. Prior studies of pacemakers in patients undergoing MRI procedures have provided groundwork for design improvements. Factors related to magnetic field interactions and transfer of electromagnetic energy led to specific design changes. Ferromagnetic content was minimized. Reed switches were modified. Leads were redesigned to reduce induced currents/heating. Circuitry filters and shielding were implemented to impede or limit the transfer of certain unwanted electromagnetic effects. Prospective multicenter clinical trials to assess the safety and efficacy of the first generation of MR conditional cardiac pacemakers demonstrated no significant alterations in pacing parameters compared to controls. There were no reported complications through the one month visit including no arrhythmias, electrical reset, inhibition of generator output, or adverse sensations. The safe implementation of these new technologies requires an understanding of the well-defined patient and MR system conditions. Although scanning a patient with an MR conditional device following the strictly defined patient and MR system conditions appears straightforward, issues related to patients with pre-existing devices remain complex. Until MR conditional devices are the routine platform for all of these devices, there will still be challenging decisions regarding imaging patients with pre-existing devices where MRI is required to diagnose and manage a potentially life threatening or serious scenario. A range of other devices including ICDs, biventricular devices, and implantable physiologic monitors as well as guidance of medical procedures using MRI technology will require further biomedical device design changes and testing. The development and implementation of cardiac MR conditional devices will continue to require the expertise and collaboration of multiple disciplines and will need to prove safety, effectiveness, and cost effectiveness in patient care.

## Introduction

In a previous JCMR paper entitled, "MRI in Patients with Pacemakers and ICDs: Defining the Issues", the authors discussed controversies related to imaging patients with cardiac pacemakers and implantable cardiac defibrillators (ICDs) which had not been specifically designed for the magnetic resonance imaging (MRI) environment [1]. Since that time, technological advances have led to the first generation of MR conditional cardiac pacemakers, allowing for greater patient management options as well as a new set of issues and controversies. One paradoxical effect created by this burst of medical innovation is the potential limitation of application of a technology due to the presence of another technology in the same patient. Because a significant percentage of patients with cardiac devices may potentially have indications for MRI during their lifetime [2], application of MR conditional devices is important to allowing patients access to MR scanning under well defined conditions. The engineering and implementation of these devices requires an understanding of: 1) all device/MRI interactions, 2) design issues to minimize or eliminate these interactions, and 3) patient management decision-making algorithms for their safe application. This review will discuss the development and implementation of MR conditional cardiac pacemakers and issues related to future design and implementation of other cardiac devices.

### Studies of MRI/Cardiac Pacemaker Interactions

Previous studies have identified issues laying the groundwork for design improvements to engineer so-called MR conditional devices with the MRI environment under strict conditions for the patient, the device, and MRI parameters. The term, "MR conditional" refers to an item that has been demonstrated to pose no known hazards in a specified MR environment with specified conditions of use [3]. "Field" conditions that define the MR environment include static magnetic field strength, spatial gradient magnetic field, dB/dt (time rate of change of the magnetic field), radio frequency (RF) fields, and specific absorption rate (SAR). Additional conditions, including specific configurations of the item (e.g., the routing of leads used for a neurostimulation system), may be required.

The medical literature from the pre-MR conditional cardiac device era arises from: 1) *in vitro *investigations of cardiac pacemakers and implantable cardiac defibrillators (ICDs), 2) isolated case reports, 3) retrospective series of patients with devices unintentionally or intentionally placed in the MRI environment and 4) prospective studies with defined patient and MR conditions [4-69]. Additionally, a physician initiated prospective multicenter site registry of pacemakers and ICDs using 1.5 Tesla non-thoracic scans has been initiated (The MagnaSafe Registry) [70,71]. Table 1 summarizes *in vivo *human data related to both pacemakers and ICDs. These data demonstrated certain adverse interactions of cardiac pacemakers and ICDs in the MRI environment and provided variable results regarding safety with prospective studies under defined conditions. These data led to controversy regarding study under well-defined conditions in cases where the use of MRI was essential to patient management versus the need for devices engineered to be MR conditional [1,72-79]. The issues raised by these data, however, were essential to prospective multicenter human studies completed [80-82], ongoing [83], and planned [84] to assess the safety and efficacy of the first generation of MR conditional cardiac pacemakers.

**Table 1:** MRI in Patients with Pacemakers and ICDs.

Author	Device	Year	Patient/Studies Report Type	MRI Condition	Findings
Iberer [8]	PPM	1987	1/1 Case		No adverse effect

Alonga [11]	PPM	1989	1/1 Case Intentional	1.5 T Brain	No adverse effect

Inbar [12]	PPM	1993	1/1 Case Intentional	1.5 T Brain	No adverse effect

Gimbel [15]	PPM	1996	5/5 Retrospective Intentional	0.35-1.5T Cardiac, Brain, C-Spine	Two second pause

Garcia-Boloa [16]	PPM	1998	1/2 Case Intentional	1.0 T Brain	No adverse effect

Fontaine [98]	PPM	1998	1/1 Case Intentional	1.5 T Brain, C-Spine	Rapid pacing

Sommer [143]	PPM	1998	18/18 Prospective	0.5 T Brain, Cardiac, Vascular	Asynchronous mode due to activation of the reed switch in all patients

Sommer [18]	PPM	2000	45/51 Prospective	0.5 T Multiple	No adverse effect

Valhaus [20]	PPM	2001	32/34 Prospective	0.5T Multiple	Decrease in battery voltage recovered at 3 months

Martin [29]	PPM	2004	54/62 Prospective	1.5 T Multiple	Significant change in pacing threshold in 9.4% of leads, and 1.9% of leads requiring an increase in programmed output.

Del Ojo [32]	PPM	2005	13/13 Prospective	2.0 T Multiple	No adverse effect.

Rozner [41]	PPM	2005	2/2 Case Intentional	1.5 T Thorax, Lumbar	Transient change to ERI in 1 patient.

Gimbel [33]	PPM	2005	10/11 Prospective	1.5 T Brain C-Spine	Small variances in pacing threshold were seen in four patients.

Sommer [50]	PPM	2006	115/82 Prospective	1.5 T Extra-thoracic	Significant increase in pacing threshold, decreased lead impedance, and decrease in battery voltage. No inhibition of pacing or arrhythmias and no leads which required an increase in pacing output.

Heatlie [52]	PPM	2007	5/6 Prospective	0.5 T Cardiac	Pacing at maximum voltage at a fixed rate of 100 beats/minute in one patient.

Anfinsen [22]	ICD	2002	1/1 Case Inadvertent	0.5 T Brain	Inappropriate sensing, battery voltage transient change to EOL.

Fiek [28]	ICD	2004	1/1 Case Inadvert	0.5 T Brain	Unable to communicate with device.

Coman [30]	ICD	2004	11/11 Prospective	1.5 T Cardiac, Vascular, General	Brief asymptomatic pause in 1 patient. Unable to communicate with device in 1 patient.

Gimbel [34]	ICD	2005	7/8 Prospective	1.5 T Brain, L-Spine	"Power on reset" electrical reset requiring reprogramming in 1 patient.

Roguin [39]	ICD	2005	1/1 Case Intentional	1.5 T Cardiac	No adverse effect.

Wollmann [44]	ICD	2005	1/3 Case Intentional	1.5 T Brain	No adverse effect.

Naehle [46]	ICD	2006	1/1 Case Intentional	1.5 T Brain	No adverse effect.

Nazarian [47]	PPM 31 ICD 24	2006	68/55 Prospective	1.5 T	No adverse effect.

Nemec [48]	ICD	2006	1/1 Case Unintentional	Not specified Brain	Noise detected as ventricular tachycardia and ventricular fibrillation, with no therapy presumably due to magnetic mode activation. Asynchronous pacing due to noise-reversal mode.

Sardanelli [144]	PPM	2006	1/1 Case Intentional	1.5 T Breast	No adverse effect.

Mollerus [56]	PPM 32 ICD 5	2008	37/40 Prospective	1.5 T Truncal, non-truncal	No adverse effect. No changes in cardiac troponin-I

Naehle [57]	PPM	2008	44/51 Prospective	3.0 T Brain	No adverse effect. No changes in cardiac troponin-I (Use of transmit-receive head coil)

Gimbel [59]	PPM	2009	1/1 Case Intentional	2.0 T Brain	Asystole

Goldsher [60]	PPM	2009	1/1 Case Intentional	1.5 T Cervical	No adverse effect. Scan one day after implant Pacemaker dependent

Mollerus [61]	PPM 46 ICD 6	2009	52/59 Prospective	1.5 Truncal, non-truncal	MRI-related ectopy 7 pt

Naehle [62]	PPM	2009	47/171 Case Intentional	1.5 T General	Statistically significant but clinically irrelevant change in pacing capture threshold and battery voltage. 2 or more serial scans.

Pulver [64]	PPM	2009	8/11 Prospective	1.5 T Cardiac, non-cardiac	No adverse effect. Congenital heart disease with 9 epicardial leads.

Strach [67]	PPM	2010	114/114 Prospective	0.2 T General	No adverse effect

Millar [66]	PPM	2010	1/1	1.5 T Brain C-spine	No adverse effects

Burke [117]	PPM 24 ICD 10 CRT ICD 4	2010	38/92 Prospective	1.5 T Brain, Spine, Pelvis, Extremity	No adverse effects No changes defibrillation threshold (ICD)

Buendia [100]	PPM 28 ICD 5	2010	33/33 Prospective	1.5 T Cardiac Brain, Spine, Abdominal, Extremity	Temporary communication failure in two patients. Sensing errors during imaging in two patients., Safety signal generated in one pacemaker at the maximum magnetic resonance frequency and output level.

Cohen [70]	PPM 74 ICD 31	2010	105/105 Prospective	1.5 T	No deaths, device failures, genrerator/lead replacements, loss of capture, or electrical reset. Decrease in battery voltage of ≥ 0.04 V in 2% Lead impedance change ≥ 50 Ohms in 3% High voltage impedance change ≥ 3 Ohms in 10% Decrease in R wave amplitude in 2% Pacing threshold increase of ≥ 0.5 V at 0.4 ms in 1% of leads

Wilkoff [81]	MR ConditionalPPM	2011	226/226 Prospective	1.5 T Brain and lumbar	No adverse effect

Quarta [140]	MR ConditionalPPM	2011	1/1 Prospective	1.5 T Brain, Cardiac	No adverse effect

Adapted and Updated from Shinbane et al. 2007. [1]

Case = case report, EOL = end-of-life, ERI = elective-replacement indices; ICD-implantable cardiac defibrillator; PPM = pacemaker; T = Tesla,.

In a randomized, unblinded, two arm study of patients who met standard criteria for dual chamber pacing (484 enrolled, 464 with successful implant, 258 randomized to a single non-medically indicated MRI scan and 206 randomized to a control group) there were no significant changes in pacing parameters (sensing, threshold, or impedance change) compared to controls [81]. Both pacemaker-dependent and non-pacemaker dependent patients were studied, with MR scanning in the asynchronous mode (n = 158), and no pacing (n = 67) with a continuous stable rhythm during scanning. Although pacemaker dependence was not assessed at the time of scanning, 16 MR scanned and 11 control patients had no underlying intrinsic ventricular rhythm at the pre-9- to 12-week assessment. There were no reported complications through the one month visit including arrhythmias, electrical reset, inhibition of generator output, or adverse sensations.

The safe implementation of new MR conditional technologies requires a detailed understanding of the MR nomenclature as it relates to specific patient and scanning conditions [3]. The American Society of Testing Materials (ASTM) designates devices as MR safe, MR conditional, and MR unsafe [85]. As an MR safe device would require nonmetallic, non-conducting materials and systems with no known hazards in all MR environments, it would be impossible to develop a pacing system with this designation. As MR conditional refers to devices which pose no known hazards when applied with specific conditions and a specific MR environment, the approval of a device requires strict definition of these conditions. Recently, the United States Food and Drug Administration (FDA) approved an MR conditional pacing system, the Revo™ MRI SureScan^® ^Pacing System (Medtronic, Inc., Minneapolis, MN) [81]. Outside of the United States [Biotronik (Berlin, Germany), Medtronic, Inc. (Minneapolis, MN), and St. Jude Medical (St. Paul, MN), there are commercially available MR conditional cardiac pacemakers [86,87]. Importantly, each MR conditional system is composed of an MR conditional pulse generator, MR conditional leads, and MR conditional programming (Table 2). These and other manufacturers have systems in development and testing. Each system has its own individual conditions which must be adhered to. Therefore, knowledge of the individual system conditions is essential for the safe performance of MR scanning. In reference to scanner type, the currently approved field strength is 1.5 Tesla, and scanning is not approved for field strengths above or below this strength. Due to rapid advances in technology and clinical studies, the medical staff involved in this field must continually update their knowledge base related to MR conditional cardiac devices. This includes information on changes in devices and MR conditions as specified by the approving agency in the country where the device is being implanted and patient scanned.

**Table 2:** MRI Patient and Scanner Conditions for Clinically Released MR Conditional Systems in the United States or the European Community.

System	Medtronic EnRhythm MRI™ SureScan™ Pacing System	St. Jude Medical™ MR Conditional Pacing System	Biotronik ProMRI™ MR Conditional Pacing System
**Approval**	United State (FDA appoval) European Community (CE approval)	European Community (CE approval)	European Community (CE approval)

**Studies**	Wilcoff et al. [81] Forleo et al. [82] Advisa MRI Study [83]. (In process)	Accent MRI Study [145]. (In process)	

**Implant site**	Left or right pectoral region	Left or right pectoral region	Chest area. Patient's height at least 1.4 meters

**Limitations related to other devices/leads**	No active or abandoned medical devices, leads, lead extenders or adaptors	No abandoned cardiac hardware including leads, lead extenders, or lead adaptors	No other pacemakers or ICDs, leads no longer in use, lead adapters, lead extension

**Implant timing prior to MRI**	At least six weeks	Stable pacing capture threshold values	At least 6 weeks.

**Acceptable lead parameters for MRI**	Capture thresholds of ≤ 2.0 V at a pulse width of 0.4 ms Impedance of ≥ 200 and ≤ 1500 Ohms No diaphragmatic pacing at 5.0 V at a pulse width of 1.0 ms	Capture threshold values of ≤ 2.5 V at 0.5. ms pulse width Lead impedance measurements within the programmed lead impedance limits No diaphragmatic stimulation at a pacing output of 5.0 V or 7.5 V and at a pulse width of 1.0 ms if device will be programmed to an asynchronous pacing mode when MRI Settings are enabled	Capture threshold ≤ 2.0 V at 0.4 ms pulse width Lead impedance is between 200 and 1500 Ohms • Battery charging status: at least 30%

**Programming for MRI**	MR conditional programming modes	MR conditional programming modes	MR conditional programming modes

**Device Identifiers**	Pulse generator radiopaque marking with a unique symbol and three letter code Unique radiopaque lead helix design	A radio-opaque MRI symbol is present on all implanted St. Jude Medical™ MR Conditional pacing system components Radiopaque MR Conditional lead marker	Pulse generator radiopaque marking. No lead radiopaque identifier

**Scanner**	1.5 Tesla cylindrical bore MR system	1.5 Tesla horizontal closed bore MR system	1.5 Tesla cylindrical bore MR system

**Landmark isocenter of RF coil**	United States: Superior to C1 or inferior to T12 Ensura MRI™ SureScan™ Pacing System and Advisa DR MRI™ SureScan™ pacemaker labeling has no restrictions on chest scans outside the United States	Contraindication to use of local transmit-only coils or local transmit and receive coils placed directly over the pacing system	Maximum allowed positioning mark for the isocenter starting from the foot at the hip bone level. and maximum allowed positioning mark for the isocenter from the top of the skull at the level of the eyes).

**Patient Positioning**	Contraindication to lateral decubitus patient positioning	Must not be positioned on side	Dorsal position only.

**Scanner Mode**	Scanner in' the normal operating mode (defined as the mode of operation of the MR system in which none of the outputs have a value that cause physiological stress to patients)	Scanner in the normal operating mode or First Level Controlled operating mode	The overall MR scanning time accumulated from the imaging times as displayed by the MRI scanner must not exceed 30 minutes The total accumulated length of examination for the pacing system must be below 10 hours

**Radiofrequency Energy**	Maximum gradient slew rate ≤ 200 T/m/s per axis Whole body specific absorption rate (SAR) levels ≤ 2 W/kg Head SAR ≤ 3.2 W/kg	Maximum gradient slew rate ≤ 200 T/m/s per axis Whole body specific absorption rate (SAR) ≤ 4.0 W/kg Head SAR ≤ 3.2 W/kg	Maximum gradient slew rate ≤ 200 T/m/s per axis Whole body specific absorption rate (SAR) ≤ 2.0 W/kg Head SAR ≤ 3.2 W/kg

### Design and Engineering Issues

A cardiac pacemaker system is composed of leads and a pulse generator composed of connectors, circuitry, and a battery. In regard to the MRI environment, factors related to magnetic field interactions and transfer of electromagnetic energy must be taken into account with the specific design of these components.

### Pacemaker Design and Magnetic Force Issues

The presence of ferromagnetic content in the strong static and gradient magnetic fields of the MR system can lead to movement and vibration of the cardiac device. These forces are directly related to the amount and shape of the ferromagnetic content, the location of this content in relation to the MR system, and the strength of the static magnetic field [88]. Although excessive magnetic field interactions can theoretically cause device movement, prospective data on both "standard" and MR conditional pacemakers at 1.5 Tesla did not demonstrate significant clinical effects [32,57,81].

One pacemaker design mechanism to limit magnetic field interactions is minimization of ferromagnetic content of the generator. Reduction of ferromagnetic content can be achieved through use of non-ferromagnetic conductive substances in the pacemaker generator. The leads are made of nonmagnetic materials. There are significant limitations on the choice of non-ferromagnetic materials, as the materials must be appropriately conductive, durable, and biocompatible.

An MRI-related factor which can decrease magnetic field interactions is the use of a lower magnetic field strength. Another factor is the maximization of the distance between the cardiac device and scanner during imaging. An example of this is a dedicated extremity MR system to scan the patient [17]. The further development of specialized scanners that utilize these factors may be useful [17,89,90].

An additional magnetic field interaction is related to the effect of a static magnetic field of the MR system on the pacemaker reed switch. The reed switch is a feature in many pacemakers designed to program a pacemaker by means of a magnet placed over the device. Reed switch magnet responses in pacemakers may cause changes to a continuous asynchronous pacing mode (DOO or VOO) in order to avoid electromagnetic interactions during such procedures as surgery with the use of electrocautery (i.e., which generates electromagnetic interference) [91]. Reed switch activation associated with exposure to an MR system may not be predictable. It may vary with strength of the static magnetic field and with orientation of the reed switch to the magnetic field [14,26,36]. Given this unpredictability, an MR conditional pacemaker has been designed with replacement of the reed switch with a solid state Hall sensor [80,81]. The Hall sensor has a more predictable behavior when exposed to magnetic fields. Another pacemaker has a magnet detect sensor that prevents problems with the reed switch [92]. Future design related to magnetic field will need to also address higher field strengths [57,59,93].

### Pacemaker Design and Electromagnetic Energy Conduction Issues

Electromagnetic energy from the MR scanner can conduct through the pacing system or cause electrical interference with components of the system. Sources of this electromagnetic energy include the pulsed radiofrequency energy from a head or body coil and the time varying magnetic fields used by the MR system for spatial localization of signals. The transfer of radiofrequency energy to heat and electrical energy is dependent on factors including: 1) the pulse sequence parameters, 2) the whole body averaged and local specific absorption rates (SAR) associated with a given sequence, 3) spatial relation and orientation of the anatomy to the transmit RF coil, and 4) lead factors (composition, length, geometry, configuration, and orientation) [51,54,55,63,94-97].

Pacing leads can potentially act as antennae for electromagnetic energy impulses [27,98]. The transfer of electromagnetic energy can lead to myocardial electrical stimulation, tissue destruction at the lead tip/endocardial interface, pain, and damage to the pulse generator circuitry/battery. This may produce adverse effects on sensing, pacing thresholds, and lead impedances, and can cause inappropriate pacing acceleration or inhibition, and battery depletion [4,6,7,9,20,29,31,33,36,41,43,50,52,61,62,98-100] In a recent investigation by Wilkoff et al. [81] on an MR conditional pacing system, these adverse effects were not observed. Pacing thresholds did not change significantly between patients with the device who were scanned and those who served as control patients.

Factors involved in pacemaker lead design relate to avoidance of the resonant frequency as well as the lead length, configuration, and position. As a lead can act as a receiver of electromagnetic impulses, avoiding the resonant frequency of a lead is of extreme importance. A resonant lead length has been associated with a greater heating effect [101].

A pacemaker lead is composed of an outer and inner insulation and an outer and inner lead coil. The lead coil is arranged in a configuration to maximize energy efficient conduction while maintaining flexibility, durability and minimization of lead diameter. The inner coil is made of filaments wound in a three-dimensional relationship coiled with a certain pitch. This has implications for MRI-related energy conduction based on the resonant frequency of the lead [35].

An example of lead design changes in an MR conditional lead is a lead modification due to a change in the pitch of the inner coil [102]. The inner coil was designed with a decreased number of coiled filars, increasing the number of winding turns and, therefore, increasing the lead inductance. This geometry limits the radiofrequencies that can conduct through the lead filaments. The decrease in the number of filars required an increase in the filar diameter to increase the strength of the lead. Additionally, a lead tip coating was used with a substance that decreased polarization. As unipolar pacing is more susceptible to the MRI environment, a bipolar lead design was used so that the generator was not essential to the pacing circuit [21,43].

Measurements of lead heating with in vitro models can be challenging [103]. In models, many factors affect heating at the lead tip including length and the geometric structure of the lead, phantom shape, and position of the radiofrequency coil. Closer proximity of leads to the edge of the phantom and to the edge of the coil caused greatest heating [55]. Another phantom study at 1.5 T demonstrated greatest heating with the torso centered along the superior-inferior direction of the transmit coil [97]. In vitro study of lead design has demonstrated increased heating with increased lead insulation thickness and uncoiled leads of 25-50 cm length [104]. This study demonstrated decreased heating with increased lead impedance and winding configuration with reversed winding segments.

Abandoned leads can also conduct radiofrequency energy. In an *in vivo *study of clinical lead lengths (40-60 cm), abandoned leads demonstrated greater lead tip heating in comparison to leads attached to pulse generators [68]. Therefore, no additional abandoned leads should be present [81]. As interactions can occur with abandoned leads, special attention to the patient's device history regarding abandoned leads is important.

Circuitry filters to impede or limit the transfer of certain frequencies, circuits that divert and dissipate energy, and generator shielding can also be important to design in order to avoid or minimize the transfer of electromagnetic energy. Of note, retained epicardial wires cut short at the skin level from previous cardiothoracic surgery procedures do not appear to be associated with serious MRI-related issues [105,106].

### Application of Defined MR Conditions

Of paramount importance to the implementation of these new technologies is the education and training of the medical community involved in care of cardiac device patients who are undergoing MR scans. The goals of education are to provide optimal patient care and efficient patient flow in the MR center. This includes understanding of the pacemaker programming to an MR conditional mode. The design of the programming hardware and software must include features to inactivate sensing, internal assessment of system integrity, and clear programming return to pre-scan values.

**Figure 1:**
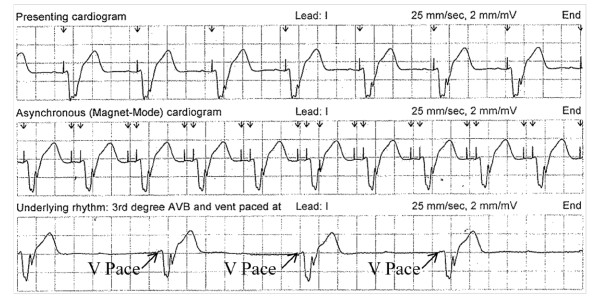
**Intracardiac Electrocardiograms Demonstrating Pacemaker Dependence**. Upper Panel: The presenting rhythm is sinus rhythm at 80 beats per minute with ventricular pacing. Middle Panel: Asynchronous dual chamber pacing. Lower Panel: The underlying rhythm is sinus with complete heart block with ventricular pacing at 35 beats per minute. This patient has complete heart block with no ventricular escape at 35 beats per minute.

The decision as to the appropriate pacing mode during MR scanning requires arrhythmia expertise. Understanding of the patient's initial indication for the device, arrhythmia history, and underlying rhythm/pacemaker dependence are important aspects of clinical information. The underlying sinus rate, AV nodal conduction, and presence rate and location of escape rhythms are important in making decisions as to the appropriate programming mode (Figure 1). Additionally, many pacemakers have logs listing the percentage of time that a patient is paced in the atrium or ventricle. One needs to interpret this data though in regard to the programmed pacing rate and programmed AV intervals, as the patient may have a regular rhythm under these programmed parameters. As predicting bradyarrhythmic or tachyarrhythmic events on a beat by beat basis is challenging, even when the appropriate mode is chosen based on past history, meticulous and continuous monitoring of the patient's heart rate and rhythm is required while the patient is programmed in the appropriate MR conditional mode. Asynchronous pacing is used with scanning a pacemaker dependent patient. In those patients with an underlying rhythm who are not bradycardic, a ventricular rate competing with the asynchronous pacing could lead to pacing during ventricular repolarization, potentially causing an R on T phenomenon leading to life threatening ventricular arrhythmias [36,107,108].

The pacemaker programmer device must be outside of the MR scanner room. Device programming immediately before entering the MRI environment and device interrogation and reprogramming immediately after removal from the MRI environment can limit the amount of time that the patient is in the MR conditional mode. Before MRI scanning, the initial programming mode is stored, so that devices can be programmed back to the original settings.

Even with advances in use of MR conditional ECG monitoring equipment, the MRI environment causes artifacts in the recordings. Therefore, meticulous attention to ECG electrode pad positioning and skin prep must be performed to assure adequate monitoring [109,110]. Visual and verbal assessment of the patient's level of consciousness, blood pressure and pulse oximetry monitoring provide additional mechanisms to alert the MRI technologists, nurses, and/or physician of any unusual sensations or issues. The presence of healthcare professionals who have completed training in the programming and scanning of the specific MR conditional device and availability of a defibrillator (albeit, to be used outside of the MR system room), are essential.

Although scanning a patient with an MR conditional device following the strict patient and MRI-related conditions appears straightforward, issues that impact patients with pre-existing devices remain complex. Until MR conditional devices are the routine platform for all of these devices, there will still be challenging decisions regarding using MRI in patients with pre-existing devices where imaging is required to diagnose and manage a potentially life threatening scenario, such as a central nervous system tumor, cord compression, stroke or hemorrhage [44,47,60]. The previous database for non-MR conditional devices is, therefore, still important in individualizing the risk:benefit ratio in these challenging circumstances [1,69,111,112].

As the MR conditional system was designed for the pulse generator to be used only with the MR conditional leads, an MR conditional generator cannot be simply attached to pre-existing leads and retain MR conditional labeling. A decision would need to be made as to whether to scan the patient with their pre-existing system or whether to extract the pacing system and place an MR conditional cardiac pacemaker. Because there can be significant complications with system extraction and replacement, including vascular damage, cardiac perforation, infection, etc., the risk:benefit ratio of these options needs to be carefully decided by physicians with expertise in these areas [113]. Additional questions will relate to the best approach to generator change-outs in patients at elective replacement who will likely require MRI procedures in the future, recognizing that change to a MR conditional system requires explant of the entire system including leads and implant of new system.

### Special Design Issues Related to ICDs

Data related to the use of MRI in patients with ICDs are limited with variable results, with some isolated reports demonstrating no significant effects while others described issues with ability to communicate with the ICD post scan, sensing of electromagnetic noise, and changes in battery voltage [22,28,34,39,44,46-48,53,56,61,88,100]. ICDs possess additional complexity compared to pacemakers due to the larger size and complexity of leads, circuitry related to arrhythmia detection and treatment, and capacitors for cardioversion and defibrillation [114-116].

The increased ferromagnetic content of ICDs compared to pacemakers will require special attention to design and study of magnetic field interaction issues. Magnetic field interactions with the magnet response of many ICDs may cause inactivation of arrhythmia detection leading to inhibition of ICD therapies. Reliable magnetic responses in the MRI environment will need to be assessed as well as programming to ensure that ICD antitachycardia pacing, cardioversion and defibrillation therapies are not attempted by the ICD while the patient is in the MR scanner.

With ICDs, transmitted electromagnetic currents associated with MRI could potentially trigger tachyarrhythmia detection, if detection is not programmed to the "off" mode before MRI. It is unclear, though, whether ICD capacitors can charge in the presence of the MR system's powerful static magnetic field [46]. ICD programming in the MRI environment will require reliable and clear mechanisms to deactivate sensing modes to avoid inappropriate sensing of radiofrequency energy, as well as programming of pacing modes along the lines of MR conditional pacemakers.

Special intra-scan issues related to ICDs stem from the fact that these patients typically have an underlying cardiac substrate predisposing them to potentially life threatening ventricular arrhythmias. The arrhythmia history regarding the frequency of arrhythmias and overall cardiovascular patient stability are important, and these patients require meticulous monitoring while therapies are inactivated during an MRI examination. Programming design will also need to provide straight forward mechanisms to activate and confirm the original programming post-scan. The need for post-scanning ICD defibrillation threshold testing is another issue which warrants further assessment [117].

### Future Issues Related to the Spectrum of Cardiac Devices and Scanners

Given the expanding role of biomedical devices in diagnosis and treatment of medical conditions, design and engineering will play an important role in the future of many technologies as they relate to the MRI environment [118]. The gamut of cardiac devices includes atrial/biventricular and biventricular pacemakers and ICDs, subcutaneous ICDs, arrhythmia monitors and implantable physiologic measurement devices, and temporary pacing systems. Each of these technologies has a different lead number, shape, size and location, as well as generator function, size, and geometry. Additionally, neuromodulation devices (including those devices used for vagus nerve stimulation, deep brain stimulation, spinal cord stimulation, and stimulation of other organs) derived from cardiac pacemaker technologies are being implanted in patients with potential problematic MRI interactions and, thus, the need for further design modifications [119-125].

MRI is also utilized to direct such procedures as cardiac vascular/valvular interventions, cardiac electrophysiology procedures, biopsy, surgical, ablative, cryogenic, directed chemotherapeutic procedures, and neuromodulation treatments [126-137]. Biomedical device design changes will be necessary to allow the use of these technologies when they are MR-guided to avoid device/imaging interactions [138,139].

Design and engineering will not only need to take into account safety, but also: 1) the effect of MR conditional devices on diagnostic quality of the MR images, 2) application to cardiac imaging, 3) cost effectiveness and 4) impact on overall patient care. As MRI artifacts principally affect the local region of implanted devices, for pacemakers, ICDS, and other cardiac biomedical devices, artifact issues will be most important to cardiovascular and thoracic imaging [31,39,64,140]. The currently released MR conditional pacemaker in the United States requires specific landmarking steps be taken to acquire cardiac images, although not in Europe. In the European Community, the Biotronik system has a scan exclusion zone with the maximum allowed positioning mark for the isocenter starting from the foot at the hip bone level and the maximum allowed positioning mark for the isocenter from the top of the skull at the level of the eyes, while the St. Jude system specifies contraindication only to the transmit-only or local transmit and receive coils directly over the pacing system [81,86,87].

Both isocenter and artifact issues are important to the future of cardiac imaging using MR conditional pacemakers. Regarding artifacts from the pacer components, there is no easy solution, as gradient echo and steady state free precession images may be significantly distorted by regional changes in magnetic field homogeneity. For MRI examinations involving the region of the thorax, issues related to artifacts (i.e., both signal loss and image distortion) will obviously have great importance. In one study of patients with cardiac pacemakers or ICDs, 93% of thoracic scans were diagnostically acceptable [47].

Regarding chest imaging, issues of stress testing necessitate further investigation. The underlying lack of chronotropic competence in many patients with pacemakers and well as current programming algorithms with fixed pacing rates or programmed off for MR conditional pacemakers could be challenging for studies which require an elevation to 85% of the maximum of heart rate with stress. Coronary vasodilator stress would appear to be the most practical approach, but require further study. As with many new technologies, decisions regarding implementation in populations, cost, reimbursement and continued future design systems will need to be investigated (Table 3).

**Table 3:** Issues Raised by the Advent of MR Conditional Cardiac Devices Which Require Further Study.

Patient Selection	Who should receive MR conditional devices? Should MR conditional devices become the standard platform or used only in those patients with a higher risk of needing MR scanning in the future? Who is at highest risk of needing future MR scanning?
**Medical** **Coordination**	What are the most efficient and safest algorithms for coordination of medical staff from multiple disciplines (radiology, cardiology, cardiac electrophysiology, pacemaker technicians, MR technicians), in assessing, monitoring, and scanning patients?

**Study Type and Quality**	What is the spectrum of MR studies that can be performed with MR conditional cardiac devices regarding study types, imaging sequences, and the use of stress testing? What is the impact of MR conditional devices on image quality? How can cardiac MR imaging be performed with MR conditional devices?

**Impact on Care**	Does use of MR conditional cardiac devices improve the quality of patient care? What is the optimal approach to MR scanning in a patient with a non-MR conditional device in place regarding risks of extraction and replacement versus scanning with the implanted system?

**Cost**	What is the impact of implantation of MR conditional devices on device cost, physician and technologist time and cost for assessment and monitoring of patients? How should reimbursement and reimbursement codes for system placement and peri-scanning monitoring/device assessment be determined?

## Conclusions

The advancement of MR conditional technology has led to greater options for patient management and has also resulted in greater complexity of clinical issues. The current MR conditional pacing technology provides definite solutions to some specific issues related to MR scanning. Future design, engineering, testing, and implementation of systems will need to focus on a goal of broadening these applications to further decreasing the barriers to scanning patients with cardiac and non-cardiac, electronically-activated devices. Although the current literature provides data on the first generation of MR conditional cardiac pacemakers from multi-center trials conducted at research centers, post market data will be essential to assess impact of implementation of this technology in "real world" scenarios and provide long term data on the function of these systems [102,141]. The impact of MR conditional pacemakers will also be dependent on implant rates of pacemakers versus ICDs, which vary by country [142]. Further research is required on multiple issues related to MRI interactions with devices, including, the effects of multiple scans on these systems and system effects at higher static magnetic field strengths [102]. The design, development, study and implementation of cardiovascular MR conditional devices will continue to require the expertise and collaboration of multiple disciplines and will need to prove safety, effectiveness and cost effectiveness in patient care.

## Competing interests

Frank G. Shellock is a consultant for Medtronic. Boston Scientific, Biotronik, and St. Jude Medical. Jerold S. Shinbane and Patrick M. Colletti have no conflicts of interest.

## Authors' contributions

JSS participated in writing and editing of the manuscript, providing perspective from the Cardiology standpoint. PMC participated in writing and editing of the manuscript, providing perspective from the Radiology standpoint. FGS participated in writing and editing of the manuscript, providing perspective from the MR safety standpoint. All authors read and approved the final manuscript.
